# Novel odd-chain cyclopropane fatty acids: detection in a mammalian lipidome and uptake by hepatosplanchnic tissues

**DOI:** 10.1016/j.jlr.2024.100632

**Published:** 2024-08-27

**Authors:** Hany F. Sobhi, Kelly E. Mercer, Renny S. Lan, Laxmi Yeruva, Gabriella A.M. Ten Have, Nicolaas E.P. Deutz, Brian D. Piccolo, Jean Debédat, Lindsay M. Pack, Sean H. Adams

**Affiliations:** 1 Center for Organic Synthesis, Department of Natural Sciences, Coppin State University, Baltimore, Maryland, USA; 2 Department of Pediatrics, University of Arkansas for Medical Sciences, Little Rock, Arkansas, USA; 3 Arkansas Children’s Nutrition Center, Little Rock, Arkansas, USA; 4 USDA-ARS Southeast Area, Microbiome and Metabolism Research Unit, Arkansas Children’s Nutrition Center, Little Rock, Arkansas, USA; 5 Center for Translational Research in Aging and Longevity, Department of Health and Kinesiology, Texas A & M University, College Station, Texas, USA; 6 Department of Surgery, University of California Davis School of Medicine, Sacramento, California, USA; 7 Center for Alimentary and Metabolic Science, University of California Davis School of Medicine, Sacramento, California, USA

**Keywords:** xenometabolite, xenometabolism, microbial metabolism, microbiome, microbiota

## Abstract

Microbe-produced molecules (xenometabolites) found in foods or produced by gut microbiota are increasingly implicated in microbe-microbe and microbe-host communication. Xenolipids, in particular, are a class of metabolites for which the full catalog remains to be elaborated in mammalian systems. We and others have observed that *cis*-3,4-methylene-heptanoylcarnitine is a lipid derivative that is one of the most abundant medium-chain acylcarnitines in human blood, hypothesized to be a product of incomplete β-oxidation of one or more “odd-chain” long-chain cyclopropane fatty acids (CpFAs). We deduced two possible candidates, *cis*-11,12-methylene-pentadecanoic acid (*cis*-11,12-MPD) and *cis*-13,14-methylene-heptadecanoic acid (*cis*-13,14-MHD). Authentic standards were synthesized: *cis*-11-pentadecenoic acid and *cis*-13-heptadecenoic acid were generated (using Jones reagent) from *cis*-11-pentadecene-1-ol and *cis*-13-heptadecene-1-ol, respectively, and these were converted to CpFAs via a reaction involving diiodomethane. Using these standards in mass spectrometry analyses, we determined the presence/absence of *cis*-11,12-MPD and *cis*-13,14-MHD in archived piglet biospecimens. Both CpFAs were detected in rectal contents of sow and soy-fed piglets. Archived mass spectra were analyzed post hoc from a second independent study that used tissue-specific catheterization to monitor net metabolite flux in growing pigs. This confirmed the presence of both CpFAs in plasma and revealed a significant net uptake of the odd-chain CpFAs across the splanchnic tissue bed and liver. The results confirm that the novel xenolipids *cis*-11,12-MPD and *cis*-13,14-MHD can be components of the mammalian lipidome and are viable candidate precursors of *cis*-3,4-methylene-heptanoylcarnitine produced from partial β-oxidation in liver or other tissues.

Diet patterns, specific foods, and diet supplements are linked to health outcomes and physiological regulation through mechanisms involving specific food-derived molecules and other “nonhost” metabolites generated by microbiota (xenometabolites). The characterization of specific xenometabolites that impact mammalian physiology is in its infancy, but prevalence patterns of specific gut microbiota populations have been correlated with many host health indices and functions. Examples of the latter include associations between specific intestinal microbes and immune system activities (e.g. (1, 2, 3)), secretion of gut hormones (e.g. (4)), kidney functions (e.g. (5)), amino acid metabolism (6, 7, 8), behavior, neuron and brain phenotypes (see, e.g. (9, 10, 11, 12)), and other physiological systems. Molecules generated by microbial metabolism can also serve as microbe-microbe signals that modify microbial growth, quorum sensing, and functions within the microbial community itself (13). One group of metabolites that are largely underappreciated in mammalian biology are xenolipids, which similar to mammalian lipids can be produced through biochemical reactions such as saturation, desaturation, elongation, and conjugation (reviewed in Refs. (14, 15, 16)).

Long-chain cyclopropane fatty acid (CpFA) xenolipids are products of an S-adenosyl-L-methionine-requiring microbial CpFA synthase (Cfa protein). The first long-chain CpFA to be discovered—*cis*-11R,12S-methylene-octadecanoic acid (*cis*-11,12-MOD), a.k.a. lactobacillic acid—is an even-chain lipid characterized in 1950 by Klaus Hofmann *et al.*, microbiologists studying the relationships between biotin and de novo FA production using cultures of *Lactobacillus arabinosus* (see Ref. (17)). The ensuing decade saw a series of papers demonstrating that multiple *Lactobacillus* spp. can synthesize *cis*-11,12-MOD, sometimes in amounts representing ∼30% of the total fatty acid pool, with much of the CpFA pool found in microbial phospholipids (18). The methylene group is added across sites containing double bonds, supported by studies showing ∼2–3 fold higher *cis*-11,12-MOD production by *L. delbrueckii* grown in media containing *cis*-vaccenic acid (19). Many other examples of even-chain CpFAs and cyclopropene FAs (double bond at the cyclopropane ring) have since been found in bacteria, plants, and seeds (see Ref. (18)). A Cfa from *Clostridium butyricum* was first biochemically characterized in 1964 (20, 21). Cfa relies on methionine carbon to form the cyclopropane ring, proven through Met manipulation in culture (22, 23) and using ^14^C-labeled Met (24).

Characterization of this family of lipids in the context of mammalian biology remains to be fully elaborated, but sources of CpFAs include food and gut microbiota production. The even-chain CpFAs lactobacillic acid (*cis*-11,12-MOD) and dihydrosterculic acid (*cis*-9,10-methylene-octadecanoic acid [*cis*-9,10-MOD]) are in cheeses, milk, and meats from animals consuming silage (stored plant-based feed: *cfa*-positive bacteria are resident in the silage environment) but essentially absent in those fed grasses; notably, *cis*-11,12-MOD and *cis*-9,10-MOD can be found in human blood following consumption of select cheeses (25, 26, 27, 28). *cis*-9,10-methylene-hexadecanoic acid (a.k.a. CPOA2H) is an even-chain CpFA found in a variety of foods (e.g., porcine fat, milk) and detected in human serum (∼15 μM and ∼25 μM in nonobese and obese subjects, respectively), adipose triglycerides, and heart and liver membranes (29, 30, 31). Thus, at least some CpFAs are naturally occurring components of the human lipidome.

Even less is known about odd-chain xenolipid fatty acids. A “C8:1-like” medium-chain acylcarnitine (MCAC) was detected in human blood by Hoppel *et al.* and was subsequently identified as the “odd-chain” *cis*-3,4-methylene-heptanoylcarnitine (*cis*-3,4-MHC) (32). Hoppel speculated that *cis*-3,4-MHC derives from long-chain CpFA produced by gut microbiota, noting a paper by Libert (33) reporting anecdotally that cyclopropane-containing MCACs were absent in urine from newborns and in urine from a patient treated with intravenous adriamycin (which has antibiotic properties). Notably, *cis*-3,4-MHC had the highest plasma concentration compared to other MCACs, and fasting levels were reduced coincident with improved metabolic health in sedentary insulin-resistant women with obesity after a weight loss and fitness intervention (34). In that same study, plasma *cis*-3,4-MHC was increased by acute exercise. This metabolite was also shown to have a net production from the human hepatosplanchnic bed (35). Collectively, these results are consistent with a working model (34) in which the liver is a robust consumer of as-yet unidentified “odd-chain” CpFA parent molecules that are metabolized to cis-3,4-MHC as a consequence of partial β-oxidation. This model also proposes that the long-chain CpFA parent(s) is/are initially gut derived (from food or gut microbiota) and with a fraction stored in adipose tissues and released when lipolysis is activated. As an initial step to pursue these questions, potential “odd-chain” parent CpFA precursors for cis-3,4-MHC were deduced as *cis*-11,12-methylene-pentadecanoic acid (cis-11,12-MPD) or *cis*-13,14-methylene-heptadecanoic acid (cis-13,14-MHD) by considering how sequential rounds of β-oxidation up to the cyclopropane ring could lead to cis-3,4-methylene-hepanoyl-CoA and then cis-3,4-MHC. The lack of commercial availability of most CpFAs limits studies of their sources and biology. This article describes synthesis methods to produce long-chain CpFAs, including cis-11,12-MPD and cis-13,14-MHD. The latter were used as standards in mass spectrometry analyses of pig rectal contents and blood samples as proof of principle to determine if these novel CpFAs are present in mammalian systems.

## Materials and Methods

### Materials

HPLC- and MS-grade solvents acetonitrile, methanol (MeOH), chloroform (CHCl_3_), CDCl_3_, isopropanol, anhydrous acetonitrile, chromium oxide (CrO_3_), and diethyl ether were purchased from Sigma-Aldrich (Milwaukee, WI). The N,O-Bis(trimethylsilyl)trifluoroacetamide (BSTFA) was purchased from Thermo Fisher Scientific, Inc. Unsaturated alcohol derivatives were purchased from Alfa Aesar (Tewksbury, MA) and Matreya LLC (State College, PA). MS-grade water was obtained using a Milli-Q purity system (Millipore, Billerica, MA). Solid-phase extraction columns were purchased from Bio-Rad (Hercules, CA).

### Synthesis of novel CpFAs

**Fig. 1:**
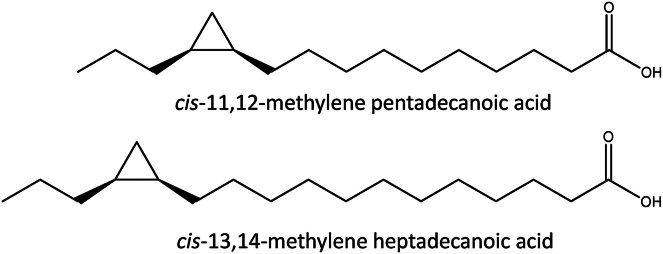
Chemical structures of the targeted long-chain cyclopropane fatty acids synthesized and characterized herein.

The long-chain CpFAs cis-11,12-MPD and cis-13,14-MHD are not available commercially, prompting development of chemical synthesis strategies to yield these compounds (Fig. 1).

### Synthesis of *cis*-11,12-MPD

This fatty acid was prepared following a protocol modified after those reported (32, 36). First, a *cis*-11-pentadecenoic acid molecule was prepared from *cis*-11-pentadecene-1-ol using Jones reagent (CrO_3_/H_2_SO_4_). In a 200 ml three-neck round bottom flask fitted with air condenser and nitrogen gas flow, *cis*-11-pentadecen-1-ol (0.4 M) was dissolved in acetone and placed in an ice bath for 10 min. Jones reagent was added dropwise over 5 min with stirring at room temperature, and the reaction mixture was then further stirred for 1.5 h. The acetone was evaporated under nitrogen gas, and the remaining solution was extracted with diethyl ether (three times, 8 ml each extraction). The combined diethyl ether fraction was further extracted with 2 ml NaOH (3.0 M), followed by acidification with 1.2 ml HCl (5.0 M) (added dropwise, tested by litmus paper). A second extraction with diethyl ether was completed, dried using 1.5 g anhydrous sodium sulfate to remove any water-soluble reactants, and the sample evaporated under nitrogen gas. The collected *cis*-11-pentadecenoic acid was then reacted with diiodomethane following a previously reported protocol (37). In a 100 ml three-neck round bottom flask fitted with air condenser and nitrogen gas flow, 15 ml of diethylzinc in hexane (1.2 M) was added while chilled in an ice-water bath, followed by additions of anhydrous benzene (15 ml), *cis*-11-pentadecenoic acid (1.20 g), and diiodomethane (2.30 ml). The solution was stirred for 5 days in a 45.0°C water bath. HCl (2.0 M) was added dropwise while stirring 10 min until acidic (by litmus paper), extracted with diethyl ether as described above, and *cis*-11,12-methylenepentadecanoic acid was collected. The *cis*-11,12-methylenepentadecanoic acid was further purified using solid phase extraction and semipreparatory liquid chromatography: *cis*-11,12-methylenepentadecanoic acid yield and purity (24% yield, 91% purity) was confirmed by ^1^H-NMR and ^13^C-NMR spectroscopy (supplemental Fig. S1).

### Synthesis of *cis*-13,14-MHD

The *cis*-13,14-methyleneheptadecanoic acid was synthesized and purified using the same procedure described above but starting with *cis*-13-heptadecene-1-ol, and the silyl ester was analyzed. Yield and purity (37% yield, 90% purity) were confirmed as described above (supplemental Fig. S2). Further analysis of the structure of the fatty acids was achieved using gas chromatography-mass spectrometry of the silyl esters. The synthesized fatty acids were derivatized using BSTFA with 1% trimethylchlorosilane (supplemental Fig. S3). The derivatization was achieved by adding 5 μl BSTFA to the synthesized fatty acid after dissolution in 1.5 ml of 98.5% dichloromethane, and the vial was vortexed for 5 min.

For both syntheses described above, the use of the classic Jones reagent (CrO_3_/H_2_SO_4_) had synergistic effects that promote the oxidation reactions selectively and significantly. We acknowledge that alternative synthesis techniques could be considered, such as microwave and ultrasound irradiation using multiwall carbon nanotube-based hybrid catalyst for the oxidation step (i.e., (38)).

### Biospecimen preparation, proof-of-principle studies for the presence/absence of “odd-chain” CpFAs

#### Study A

A subset of archived samples from piglets described in a previous study (39) were analyzed for the presence/absence of the target CpFAs. Studies were approved by the Institutional Animal Care and Use Committee of University of Arkansas Medical Sciences. Briefly, 36 Yorkshire/Duroc crossbred piglets were randomized into three groups: sow fed (sow), soy-based formula fed (soy), and cow’s milk-based formula fed (milk). Animals were fasted for 6–8 h, anesthetized with isoflurane, and euthanized by exsanguination. Samples were collected on postnatal day 21. Colon lumen fecal contents circa the rectum were flash frozen in liquid nitrogen and stored at −80°C until sample extraction. Rectal contents (50 mg; wet weight) were homogenized in 50% aqueous MeOH and extracted in acetonitrile (2:1). All samples were extracted at 4°C overnight and centrifuged at 18,000 *g* for 10 min. The supernatants were dried under a nitrogen stream and reconstituted in 5% aqueous MeOH spiked with an internal standard (Lorazepam, 500 ng/ml). Group pooled samples were prepared for each diet by mixing equal volumes of each reconstituted sample (n = 12 piglet samples used to generate a pooled representative sample per diet treatment group).

#### Study B

**Fig. 2:**
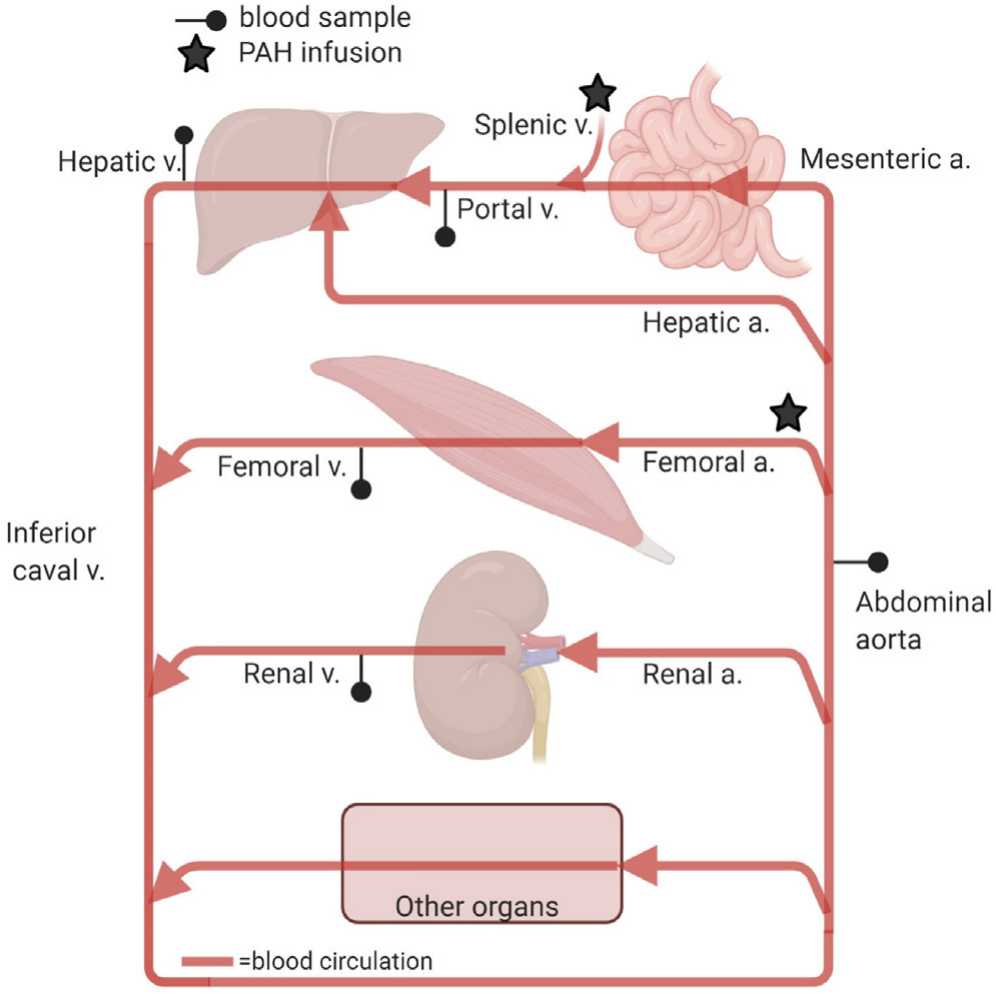
Overview of catheterization method used for growing piglets to determine the presence/absence and flux patterns of target cyclopropane fatty acids (study 2). Organ metabolite flux was measured using para-aminohippuric acid (PAH) as a blood flow marker, by placing two infusion catheters upstream of organs (*stars*). The PAH infusion site for the HQ muscle bed is the catheter in the abdominal aorta (5 cm above bifurcation) and the site for PDV and liver is the catheter into the splenic vein. Five sampling catheters (*circles*) were inserted into the following sites: (Arterial) in the abdominal aorta above the right renal artery for preorgan compartment arterial plasma concentrations; (Venous) into the inferior cava vein (iliac circumflex profunda vein) with its tip 5 cm above the bifurcation for HQ muscle flux measurements and venous concentrations; (Renal) into the left renal vein; (Portal) into the portal vein with its tip in the liver hilus; and (Hepatic) into the hepatic vein.

It is a challenge to measure portal- and liver-specific metabolite fluxes in humans, but this can be accomplished using a unique pig model that incorporates catheters to interrogate portal drained viscera (PDV), splanchnic (SPL; liver plus PDV), liver, adipose, and muscle (hindquarter [HQ]), and renal (kidney) blood. In-depth details of the surgical technique and flux calculations have been previously published (40, 41). Archived MS sample data used herein to detect the presence or absence of “odd-chain” CpFAs are from a published study of xenometabolite fluxes using this model (42). Those experiments were approved by the Institutional Animal Care and Use Committee of Texas A & M University. Briefly, pigs were fed swine feed (7200 Harlan-Teklad Vegetarian Pig/Sow Grower; Envigo, Indianapolis, IN) at 08:00 (0.5 kg) and 15:00 (0.5 kg) throughout the study and provided water ad libitum. Pigs were maintained at 21–25°C during a 12-h light-dark cycle (lights on, 07:00 and lights off, 19:00). For catheter placement, pigs were sedated (3.3 mg/kg Telazol, i.m.) after a 16 h fast (food withdrawn at 16:00 the prior afternoon), then intubated and anesthetized using isoflurane (2% in O_2_). Seven intravascular catheters were implanted (see schematic in Fig. 2) in addition to a feeding tube inserted percutaneously into the stomach. All catheters were secured in place and tunneled through the left abdominal wall, and pigs fitted with a canvas harness to protect the catheters. Catheters were kept patent with 0.5 ml of gentamycin (20 mg/ml) and α-chymotrypsin solution (225 U/ml). Animals recovered for 10–14 days prior to flux measurements.

#### Para-aminohippuric acid concentrations and flow calculations

At 08:00 h and after an overnight fast, pigs were weighed and given a primed continuous infusion of 25 mM para-aminohippuric acid (PAH) at 60 ml/h, reaching steady state (PAH_IN_ = PAH_OUT_) by 1 h. Heparinized blood samples were then taken in the following order: arterial, portal, hepatic (liver), venous, and renal, and specimens were immediately placed on ice. PAH concentrations in plasma were measured against a standard curve using a microplate spectrophotometer (43). Plasma flow through organs was calculated using the dilution of PAH over the organ compartment: Flow = (infusion rate × [PAH]_infusate_)/([PAH]_post_ - [PAH]_pre_), as described by Ten Have *et al.* (41). Plasma flow through the HQ, the PDV, and the splanchnic compartment (PDV + liver) was calculated using [PAH]_pre_ as the [PAH] in the main bloodstream sampled through line “arterial.” [PAH]_post_ is the [PAH] in the efferent vein of the target organ: line “portal” for PDV, line “hepatic” (liver) for SPL, and line “venous” for HQ. Plasma flow through the kidneys was calculated using [PAH]_pre_ as the [PAH] in the main bloodstream sampled through line “arterial” [PAH]_post_ as blood sampled through the line “renal.” For study B, total net flux for each organ site was calculated from metabolite peak areas after metabolomics data preprocessing described in the study by Mercer *et al.* (42). Total net flux, expressed as peak area/kg body weight/min, was calculated for all organ sites and then imported into the R Statistical Language (version 4.1.0) for analysis. To evaluate if flux significantly differed from zero, a one-sample Wilcoxon signed rank exact test was performed for each organ site.

### Biospecimen analysis for the presence/absence of “odd-chain” CpFAs

Chromatography was performed on a Dionex Ultimate 3000 UHPLC as using an XSelect CSH C18 reversed-phase column (2.1 × 100 mm, 2.5 μm) kept at 49°C based on a previously described method (44). Mobile phases consisted of 0.1% formic acid in water (A) and 0.1% formic acid in acetonitrile (B). The flow rate was set at 0.4 ml/min with an elution gradient as follows: 0%–1% B from 0.0 to 2.0 min; 1%–20% B from 2.0 to 6.5 min; 20%–95% B from 6.5 to 11.5 min; 95%–99% B from 11.5 to 13.5 min; 99%–1% B from 13.5–16.5 min; hold at 1% B until 20.0 min. The metabolites were directed to a Q Exactive Orbitrap mass spectrometer with data acquisition executed using Xcalibur 4.0 software (ThermoFisher Scientific). All samples were analyzed in both full scan electrospray ionization (ESI) positive and negative modes with nitrogen as sheath, auxiliary, and sweep gas set at 50, 13, and 3 units, respectively. Other conditions included resolution, 70,000 full width at half maximum; automatic gain control target, 3e6 ions; maximum injection time, 200 ms; scan range, 50–750 m/z; spray voltage, 3.50 kV; and capillary temperature, 320°C. The pooled samples were analyzed in both full scan mode as well as a data-dependent MSMS mode (ddMS2) to generate fragment profile for compound annotation in both ESI-positive and -negative modes in the following conditions: resolution, 17,500 full width at half maximum; automatic gain control target, 1e5 ions; maximum injection time, 50 ms; isolation window, 1.5 Da; and normalized collision energy 10, 30. The sample extracts were analyzed in a randomized sequence with pooled QC injected in every 10 samples. The pooled QC sample was also used for LC-MS conditioning in each matrix. The raw spectral data were processed using Compound Discoverer 3.0 and compared against the ACNC in-house library containing the synthesized CpFA spectra. The accurate mass and retention time (RT) were used in compound identification, including manual inspections of RT and MS1-MS2 mass spectra results. The software parameters for alignment were 5 ppm mass tolerance for the adaptive curve model and 2 min maximum shift for alignment. The software parameters for detecting compounds were 5 ppm mass tolerance for extracted ion chromatogram creation, 30% relative intensity tolerance for isotope search, and 3 for the signal-to-noise threshold to process centroids. The long-chain CpFAs *cis*-11,12-MPD and *cis*-13,14-MHD data presented herein were derived from ESI-positive mode.

## Results

### Mass spectra results

**Fig. 3:**
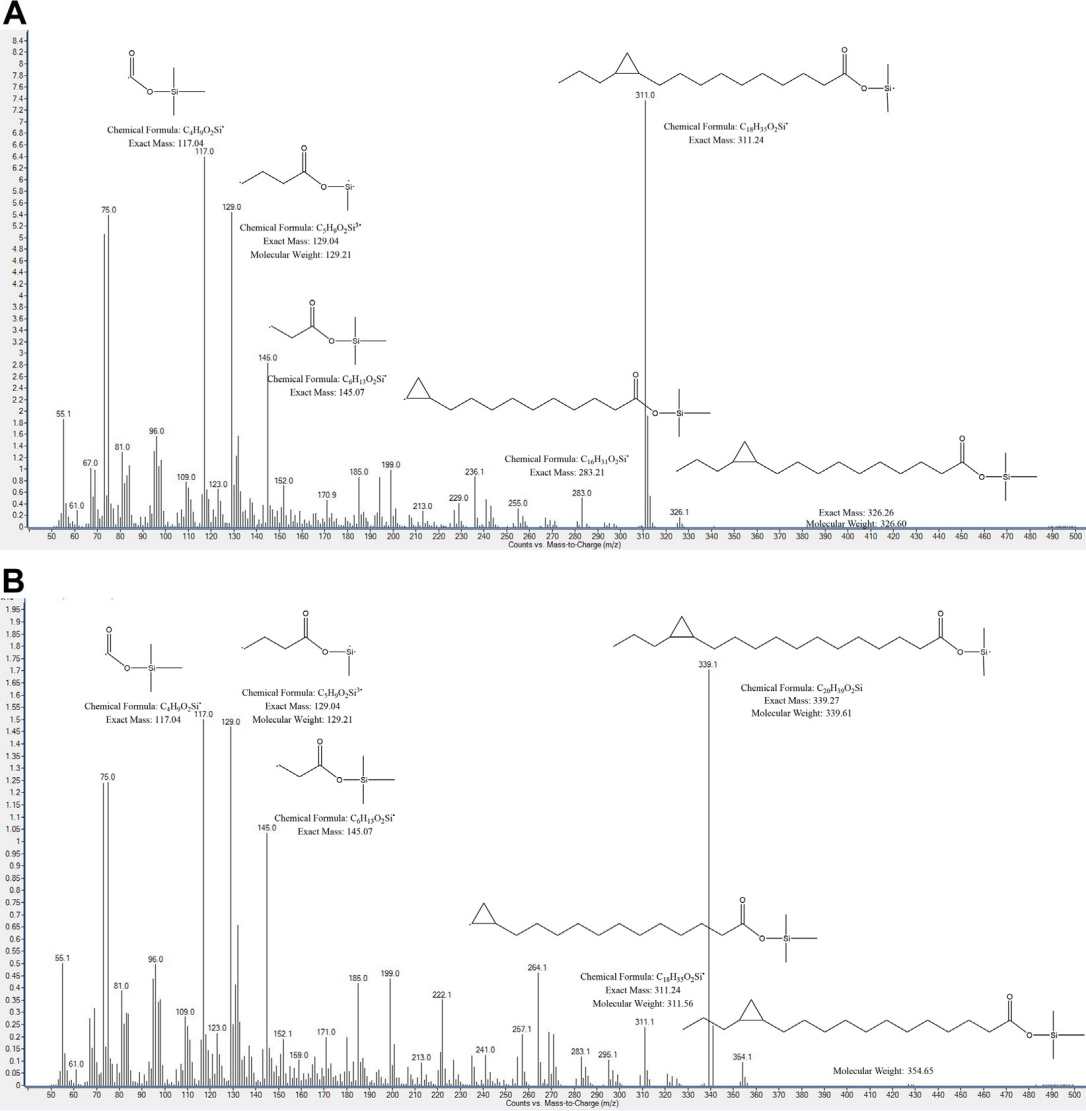
Illustrative mass spectra for derivatized (A) *cis*-11,12-methylene pentadecanoic acid and (B) *cis*-13,14-methylene heptadecanoic acid synthesized compounds. The m/z values for the parent ions (M+) were, respectively, 326.1 and 354.1.

For *cis*-11,12-MPD, the molecule has a TMS-derivatized m/z = 326.10 for the parent molecular ion (M+) (Fig. 3A). For *cis*-13,14-MHD, the TMS-derivatized M+ m/z = 354.1 (Fig. 3B).

### The presence of long-chain CpFAs *cis*-11,12-MPD and *cis*-13,14-MHD in piglet rectal contents

**Fig. 4:**
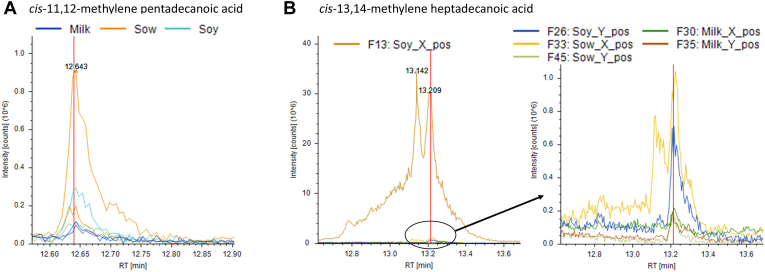
Semiquantitative identification of nonderivatized targeted cyclopropane fatty acids in biospecimens collected from sow-fed piglets or piglets fed dairy milk- or soy-based formula. The presence/absence of (A) *cis*-11,12-methylene pentadecanoic acid and (B) *cis*-13,14-methylene heptadecanoic acid was evaluated in proof-of-principle experiments (see Materials and Methods for details). Note that quantion peaks for rectal contents represent n = 6 males/diet type and n = 6 females/diet type as pools (2 pools/diet type). Thus, for rectal contents, there are two traces/diet type.

The carnitine derivative of *cis*-3,4-methylene-heptanoic acid found in human blood is thought to reflect partial β-oxidation of a long-chain CpFA (discussed in Refs. (32, 34, 35)). We deduced that potential candidates for the latter are *cis*-11,12-MPD and/or *cis*-13,14-MHD, but whether these novel “odd-chain” CpFAs exist in nature and originate in the gut have been unanswered questions. Using the newly synthesized nonderivatized *cis*-11,12-MPD and *cis*-13,14-MHD as standards to detect the presence or absence of the CpFAs by LC-MS, a proof-of-principle study was conducted in archived pooled biospecimens (rectal contents) from suckling piglets that were sow-fed or fed formula (soy or cow milk based). Both *cis*-11,12-MPD and *cis*-13,14-MHD were present in rectal contents (Fig. 4), indicating that these CpFAs are present in mammals at least in some contexts. In this sample matrix, identification of *cis*-11,12-MPD was supported by a peak in several samples at the expected RT (supplemental Fig. S4) and with predicted MS1 ion fragmentation matching the standard (not shown); however, no MS2 fragmentation data were returned from the instrument and so we consider the identification as probable (“level 2”). For *cis*-13,14-MHD, a peak was detected at the appropriate RT, and MS2 ion fragmentation patterns were confirmatory (“level 1”) (supplemental Figs. S6 and S7). In sow-fed piglets, the likely “C15” CpFA *cis*-11,12-MPD was most obviously evident (Fig. 4A), but smaller peaks were observed in the other two diet groups (Fig. 4A). Similarly, the “C17” CpFA *cis*-13,14-MHD was present in rectal contents from piglets fed all three diets, albeit with levels appearing lowest in milk formula conditions (Fig. 4B).

### *cis*-11,12-MPD and *cis*-13,14-MHD organ flux in growing pigs

**Fig. 5:**
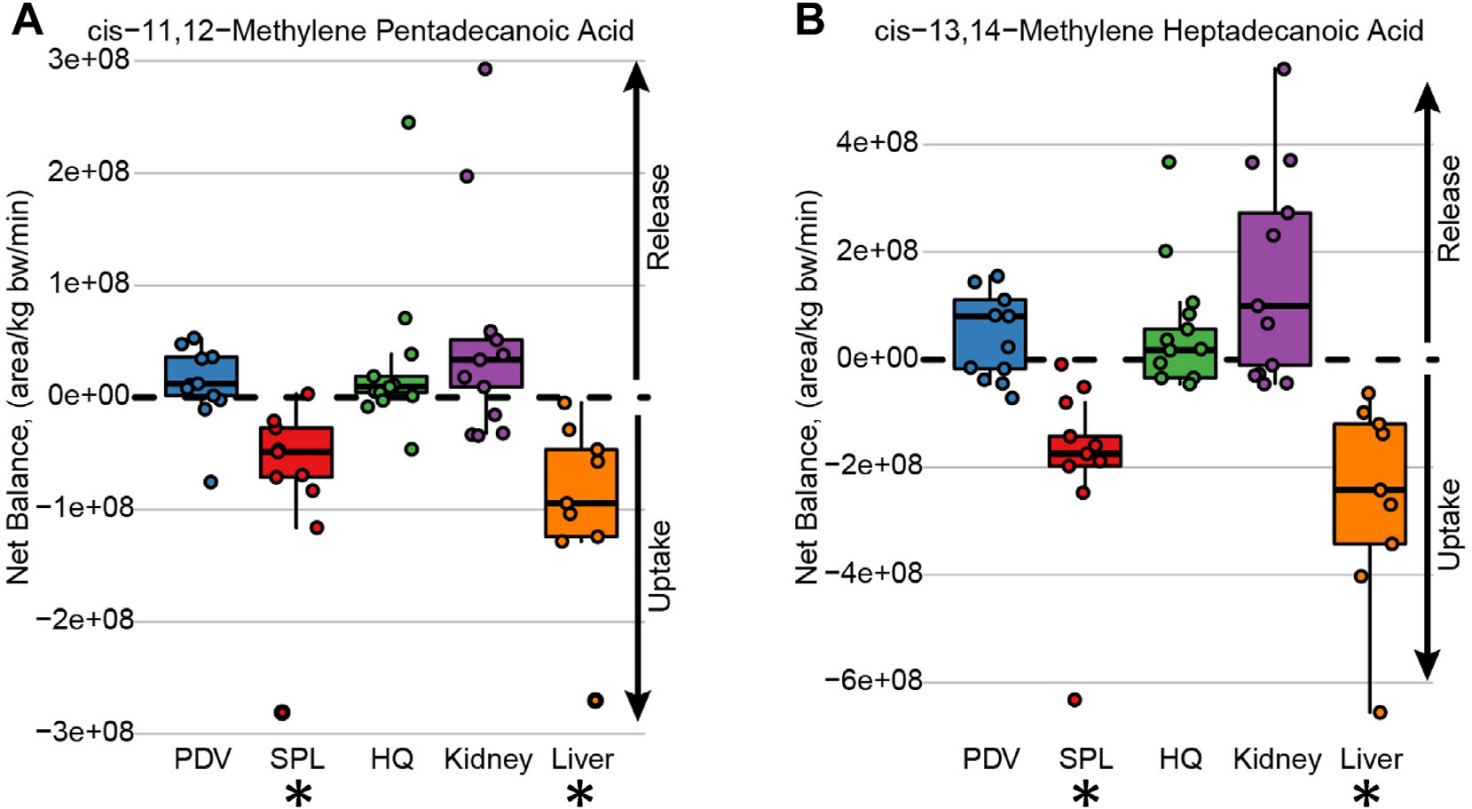
Semiquantitative abundances of nonderivatized targeted cyclopropane fatty acids in blood plasma collected from overnight-fasted growing female pigs (n = 12), fitted with strategically placed catheters to monitor metabolite fluxes in various tissues. The flux of (A) *cis*-11,12-methylene pentadecanoic acid and (B) *cis*-13,14-methylene heptadecanoic acid was determined in PDV, splanchnic tissues (SPL), HQ, kidney, and liver (see Methods for details). Concentrations used in flux calculations are quantion peak areas. ∗*P* < 0.01, net flux significantly different from zero.

Using archived mass spectra from a previously published study (42), both of the “odd-chain” target CpFAs were found to be present in plasma from growing pigs (Fig. 5), as confirmed by accurate RT and MS2 fragmentation patterns (supplemental Figs. S4–S7). Statistically significant net uptake of the long-chain CpFAs was evident for splanchnic tissues (SPL, which includes liver) and the liver proper. The flux patterns in the PDV were quite distinct compared to the splanchnic tissues (splanchnic = PDV plus liver) or compared to the liver, suggesting that at least a portion of *cis*-11,12-MPD and *cis*-13,14-MHD emanate from influx into the portal vein from the gastrointestinal (GI) tract. Flux patterns in the HQ and the kidneys were equivocal and variable across animals, and statistically, the fluxes did not differ from zero. The semiquantitative values associated with the flux studies are provided as supplemental Data.

## Discussion

Xenolipids represent microbe-derived molecules (xenometabolites) that are found in some foods and/or generated de novo by gut bacteria. The CpFA class of xenolipids have been described since the 1950s with the discovery of lactobacillic acid generated by cultures of several *Lactobacillus* species (17, 19, 45, 46, 47). Despite characterization of several CpFAs over the decades, fairly little is known about their physiology and the relative contributions of food versus gut microbial origins (see Ref. (48)). Furthermore, a comprehensive understanding of the number of CpFAs that contribute to the mammalian lipidome is lacking. Herein, we characterize, for the first time, two novel “odd-chain” CpFAs *cis*-11,12-MPD and *cis*-13,14-MHD and identified the xenolipids in mammalian biospecimens. These efforts were prompted by reports from our lab and others that cis-3,4-MHC is the most abundant MCAC in human blood (32, 34, 35, 49), which we hypothesize stems from partial β-oxidation of the “odd-chain” *cis*-11,12-MPD and/or *cis*-13,14-MHD. Indeed, early studies (e.g., tracking ^14^C-9,10-MHD or ^14^C-sterculic acid in rats) suggested that the cyclopropane group inhibits full β-oxidation of CpFAs, leading to terminal short- and medium-chain catabolic end products (50, 51, 52, 53, 54). In humans, Lindstedt *et al.* (54, 55) found a chain-shortened CpFA 3,4-methylene-hexanedioic acid as universally excreted in urine.

The synthesis of authentic standards for *cis*-11,12-MPD and *cis*-13,14-MHD is a critical first step in learning more about the origins of these long-chain CpFAs from food or de novo synthesis by gut microbes. CpFA production by mammalian tissues is not likely, based on several lines of evidence. First, using the protein sequence from the cloned *cfa* gene from *Escherichia coli* (56), a protein BLAST (blastp, National Center for Biotechnology Information) reveals no mammalian ortholog (also see Ref. (56)) despite several human methyltransferases sharing motifs (GxGG, DxxxGxG) with bacterial Cfa proteins and S-adenosyl-L-methionine-requiring enzymes from prokaryotic species. In addition to food origins for some CpFAs (i.e., (25, 26, 27, 57)), synthesis of CpFAs by gut bacteria is a strong possibility. *Lactobacillus spp.* are highly abundant in the gut, and as noted above, a number of *Lactobacillus* strains synthesize CpFAs. Furthermore, a recent cecal metabolomics survey revealed that CPOA2H was not detected in germ-free mice but appeared upon inoculation with a mixture of six microbes (58); a genome database query indicates that a subset of these (*E. coli* LF82, *Enterococcus faecalis* OG1RF, *Lactobacillus plantarum* WCFS1) carry the *cfa* gene. Additional studies are required to validate that blood and tissue *cis*-11,12-MPD and *cis*-13,14-MHD originate from the gut, but the presence of these novel CpFAs in rectal contents of sow- or formula-fed piglets supports this idea. Further evidence that *cis*-11,12-MPD and *cis*-13,14-MHD can derive from the GI tract came from post hoc analyses of blood plasma mass spectra results from growing pigs catheterized to monitor cross-tissue fluxes of metabolites, as described previously (42). Flux comparisons for *cis*-11,12-MPD and *cis*-13,14-MHD CpFAs in portal-drained viscera (neutral to net release) versus splanchnic-liver tissues (net uptake) suggest that these CpFAs could originate in the GI tract and get taken up by the liver for further metabolism, even in the overnight fasted state. The net production of *cis*-3,4-MHC, a putative medium-chain CpFA end product of *cis*-11,12-MPD and *cis*-13,14-MHD β-oxidation, from human hepatosplanchnic tissue (35), supports this idea. Additional studies will be required to refine the postprandial dynamics of CpFA influx from the gut into the circulation, including uptake as a component of chylomicrons trafficked via the lymphatics.

An intriguing, albeit preliminary, observation in sow- and formula-fed piglets was that patterns of the odd-chain CpFAs in lower GI contents appeared to differ depending upon diet type. This raises the possibility that *cis*-11,12-MPD and *cis*-13,14-MHD are components of the diet. Such a possibility could not be addressed in the current study, and it should also be noted that these diets led to significant changes in the gut microbiota communities (59), which could also have impacted CpFA patterns.

### Future directions

Building the catalog of xenometabolites found in food or those emanating from microbial metabolism of foods, environmental-, pharmaceutical-, and host-derived molecules provide a new frontier in nutrition science and physiology. The potential roles that CpFA xenolipids play in this regard remain to be established, and further work is needed to understand food versus gut microbiota origins of specific CpFAs including the novel odd-chain metabolites described herein. It is not known if fecal CpFA amounts and the presence/absence reflect patterns of CpFAs higher up on the GI tract; future studies should address bioregional differences in *cis*-11,12-MPD, *cis*-13,14-MHD, and other CpFAs. In addition, further information regarding systemic exposures to the odd-chain and even-chain CpFAs are of interest, ie, postprandially following intakes of foods containing CpFAs and following CpFA-free foods containing CpFA precursors. Another area requiring further investigation involves the precursor molecules for the odd-chain CpFAs. Very recently, Menzel *et al.* (60) reported the presence of C15:1 (11Z) (a.k.a. liebigeic acid) and C17:1 (13Z) (a.k.a. thudichumic acid) in the white waxy material (vernix caseosa) that covers babies at birth; however, these were not detected in NIST human plasma, and other biospecimens were not evaluated. Finally, since fatty acids can be incorporated into complex lipids (e.g., phospholipids, sphingolipids, ceramides, and oxylipins), it would be interesting in future studies to track if CpFAs are part of the body’s complex lipid pools (see, e.g. (29), for bovine heart phospholipids). In summary, considering the well-established importance of fatty acids and complex lipids in regulation of multiple nodes of physiology and signaling, characterization of novel xenolipids such as odd-chain CpFAs is an exciting area of discovery.

## Data availability

Data are provided as Supplemental Data and Supplemental Figures; materials and data not captured in these materials may be provided by sending a request to the corresponding authors.

## Supplemental data

This article contains supplemental data.

## Conflict of interest

S. H. A. is founder and principal of XenoMed, LLC (dba XenoMet), which is focused on research and discovery in the area of microbial metabolism. XenoMed had no part in the research design, funding, results, or writing of the manuscript. S. H. A. and J. D. are coinventors on a patent application in the area of cyclopropane fatty acids.

## Supplementary Data

Supp Figure 1A

Supp Figure 1B

Supp Figure 2A

Supp Figure 2B

Supp Figure 3

Supp Figure 4

Supp Figure 5

Supp Figure 6

Supp Figure 7
