# Supplementary figures and images for: Novel odd-chain cyclopropane fatty acids: detection in a mammalian lipidome and uptake by hepatosplanchnic tissues
Source: J Lipid Res. 2024 Aug 27;65(10):100632. doi: 10.1016/j.jlr.2024.100632 (PMC11439845; doi:10.1016/j.jlr.2024.100632)

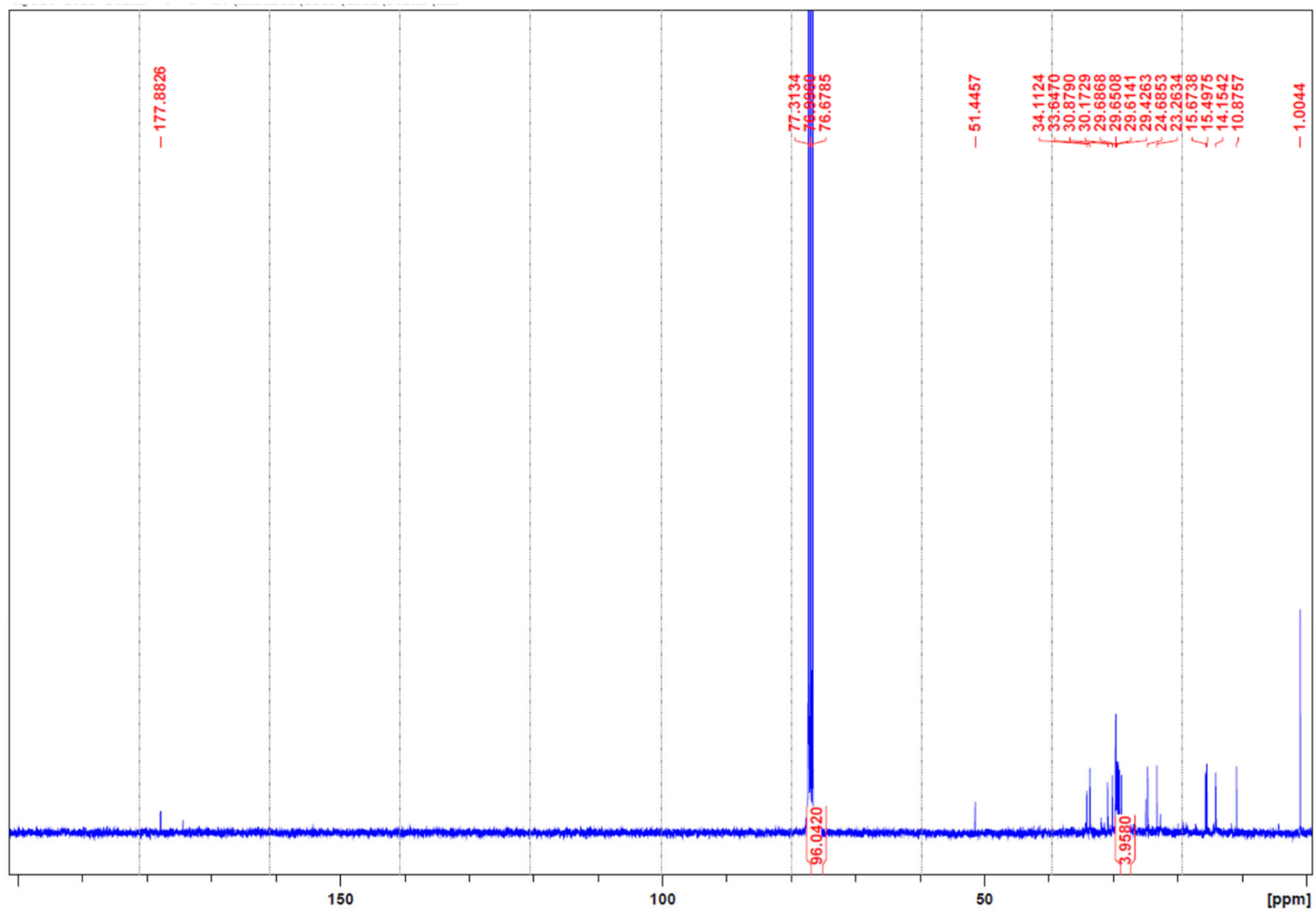

SUPPLEMENTAL FIGURE 1A

Supplement: Supp Figure 1A [file mmc1.pdf]

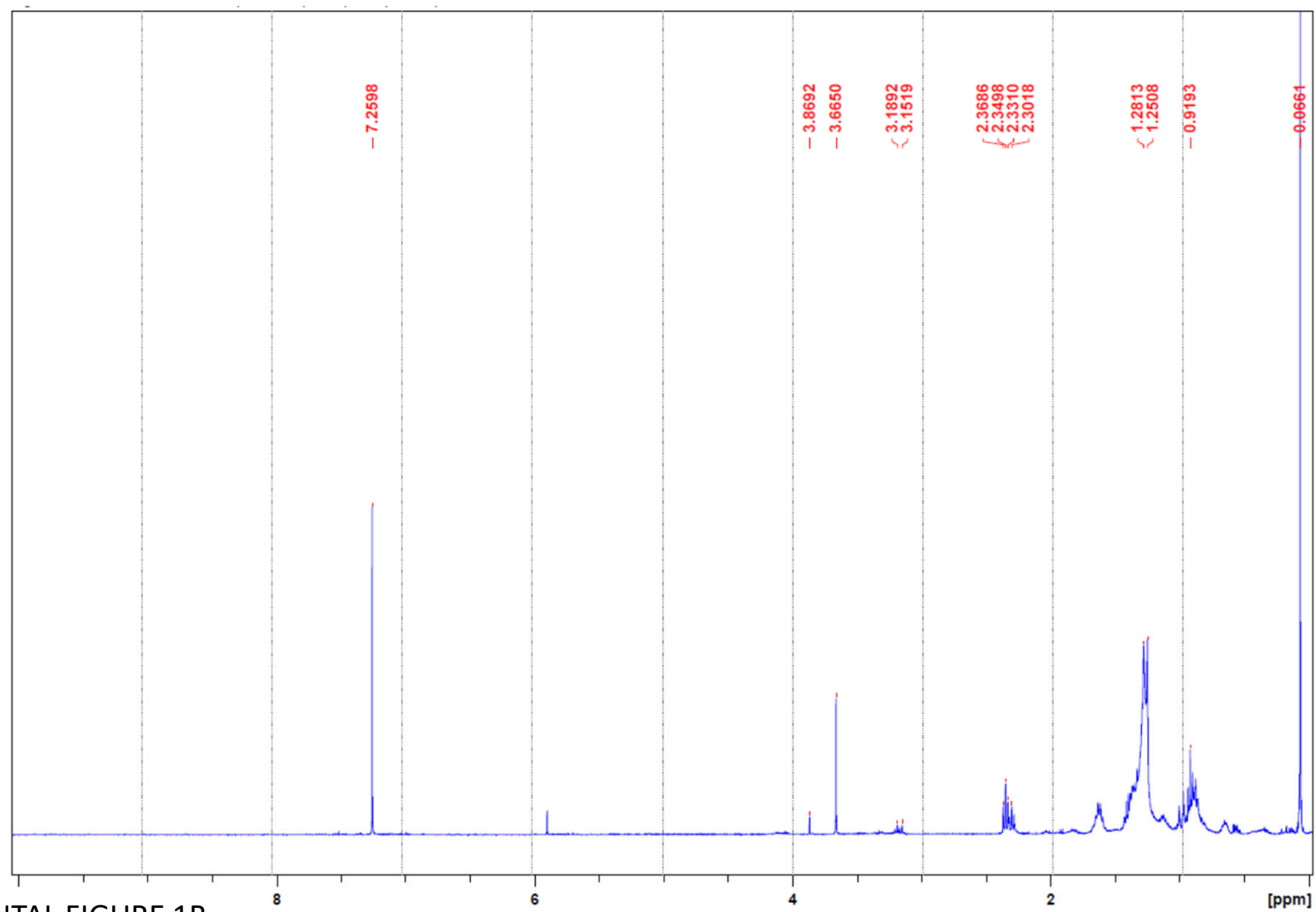

SUPPLEMENTAL FIGURE 1B

Supplement: Supp Figure 1B [file mmc2.pdf]

"C17 CpFA 20230420" 7 1 D:\nmrdata\user\data\Sobhi\nmr

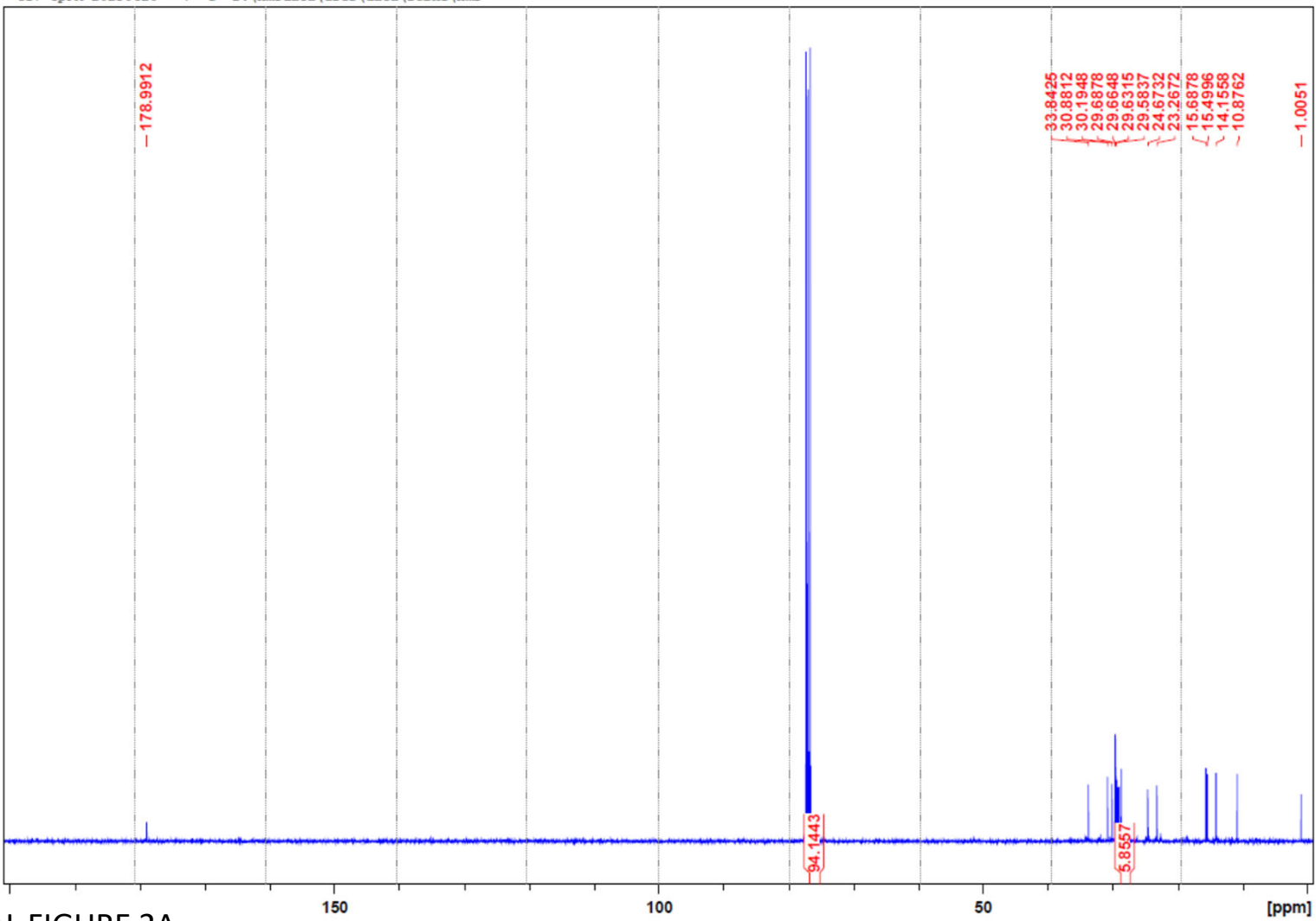

SUPPLEMENTAL FIGURE 2A

Supplement: Supp Figure 2A [file mmc3.pdf]

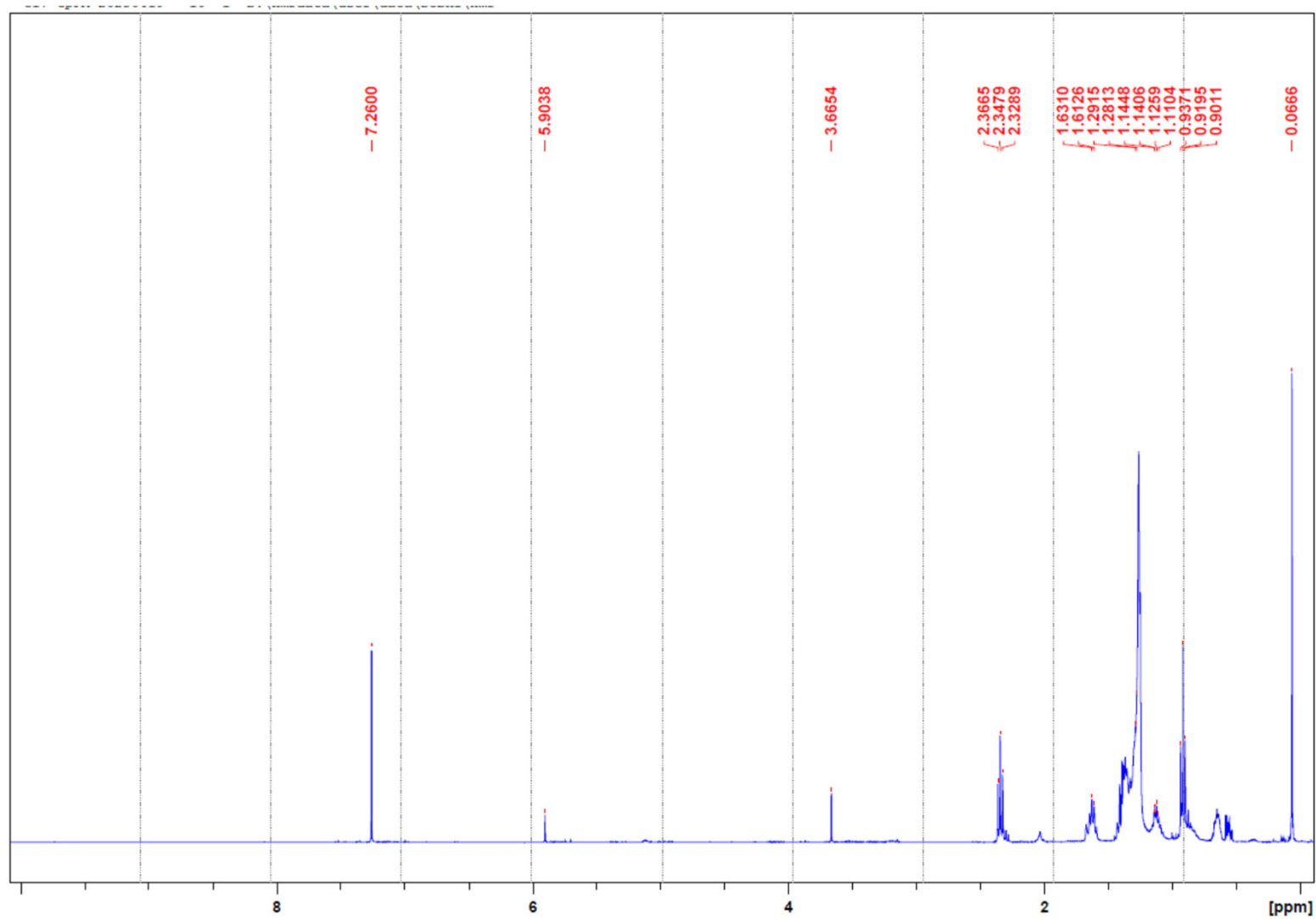

SUPPLEMENTAL FIGURE 2B

Supplement: Supp Figure 2B [file mmc4.pdf]

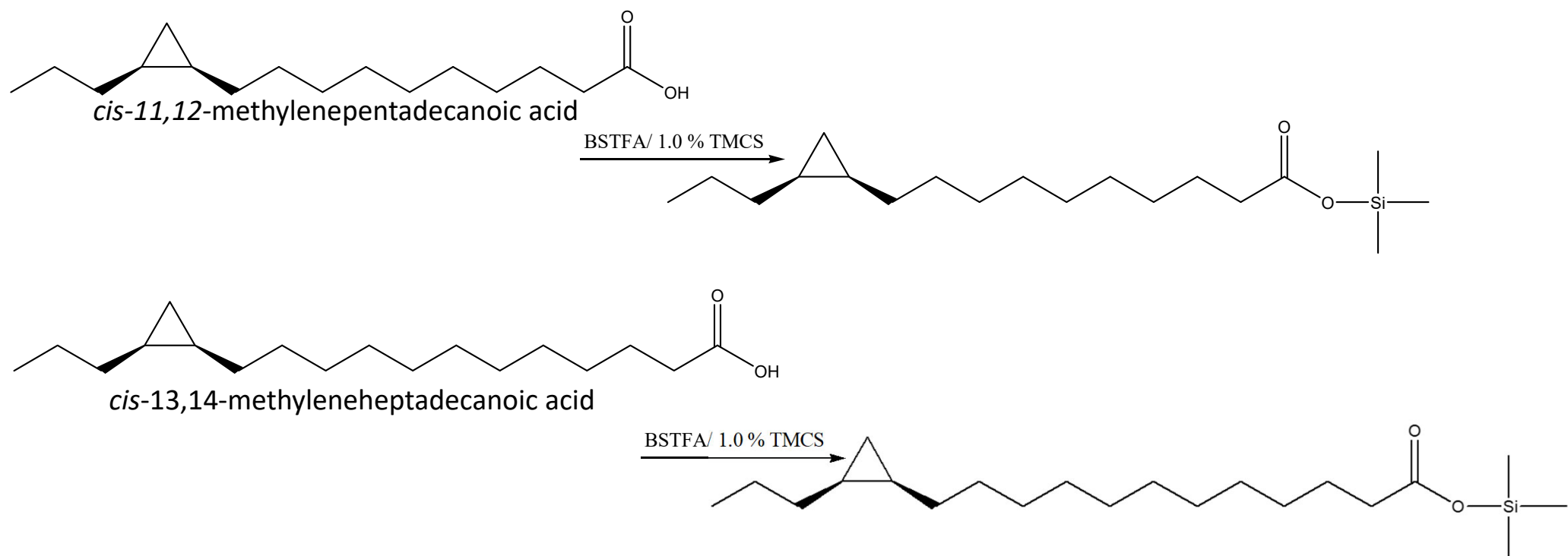

SUPPLEMENTAL FIGURE 3

Supplement: Supp Figure 3 [file mmc5.pdf]

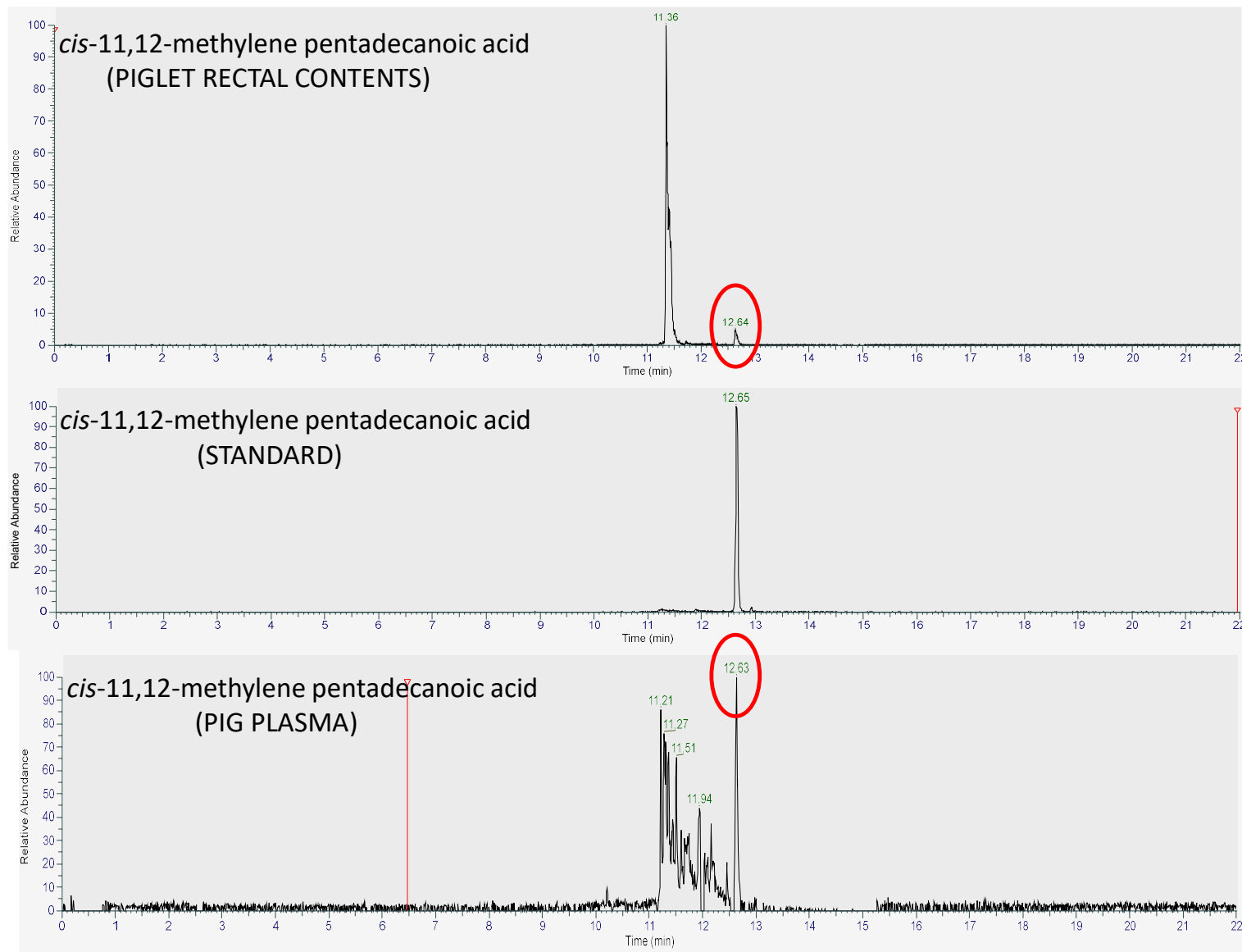

SUPPLEMENTAL FIGURE 4

Supplement: Supp Figure 4 [file mmc6.pdf]

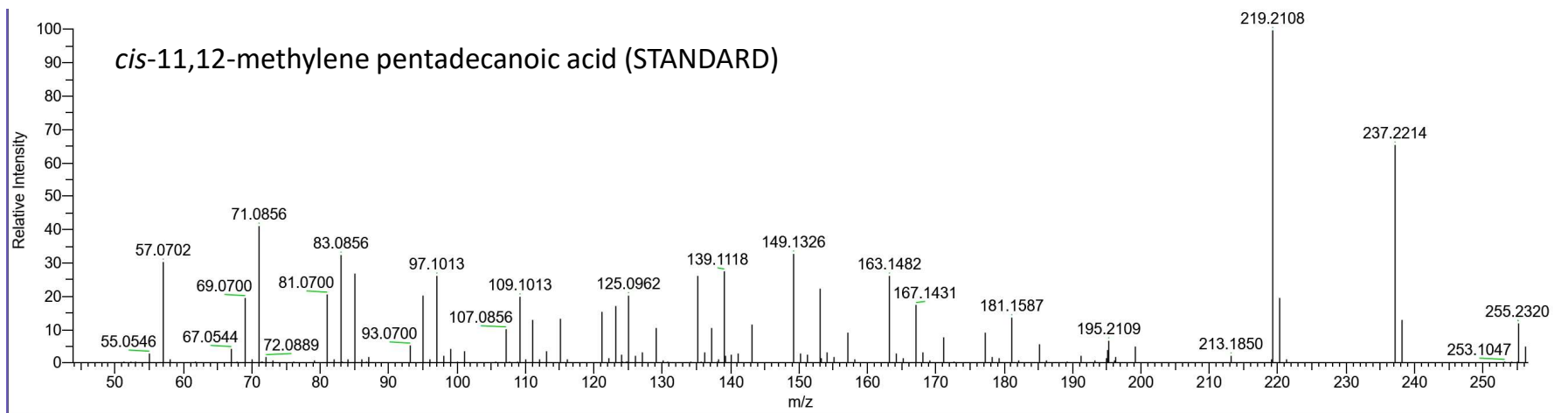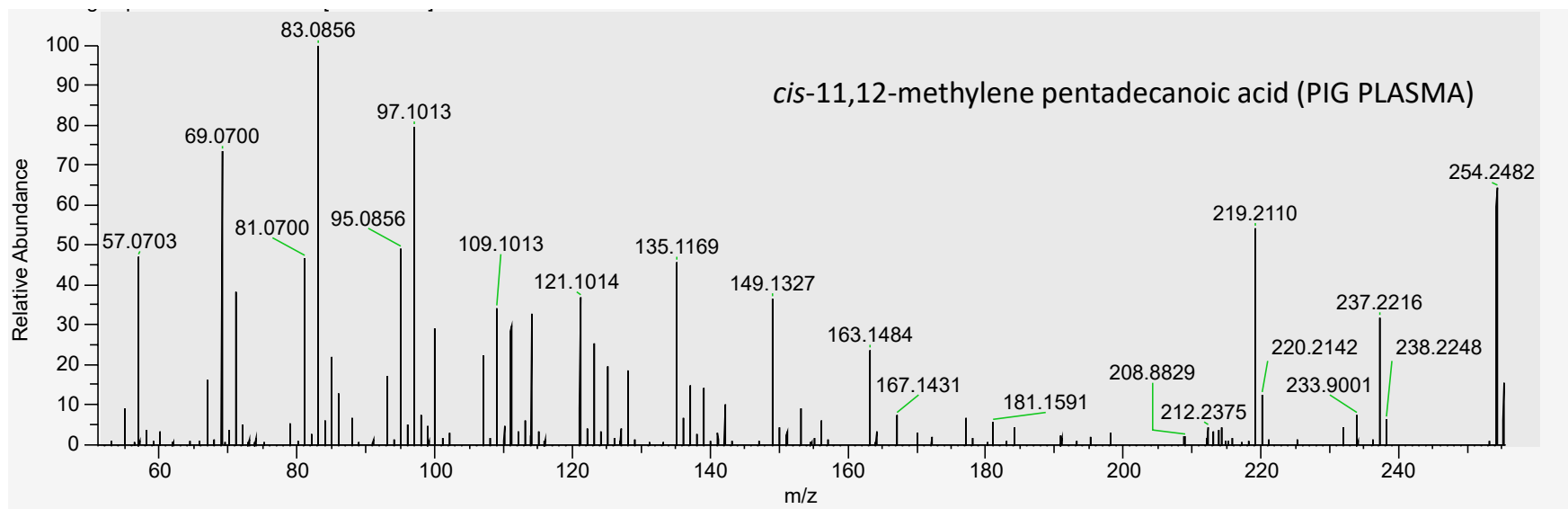

SUPPLEMENTAL FIGURE 5

Supplement: Supp Figure 5 [file mmc7.pdf]

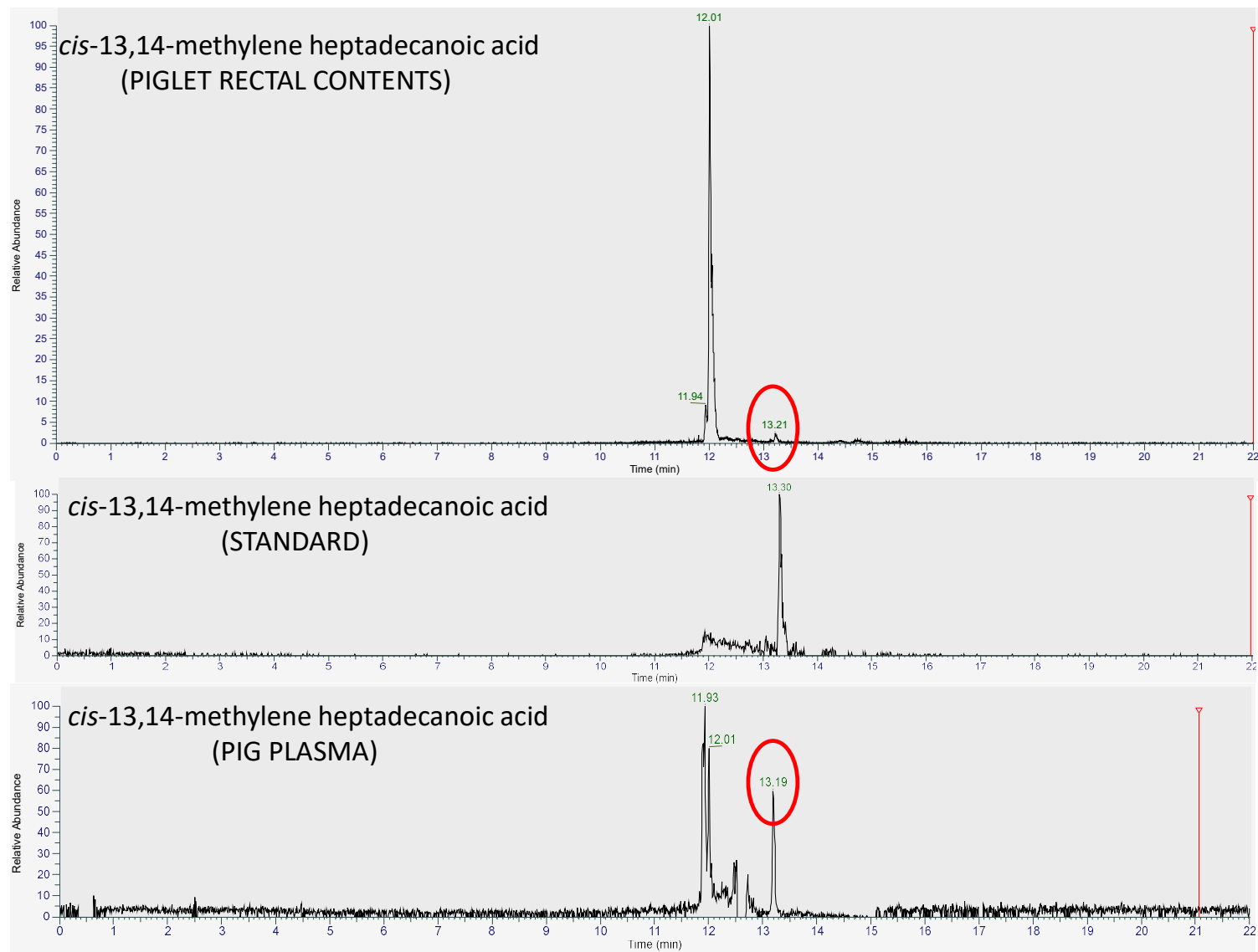

SUPPLEMENTAL FIGURE 6

Supplement: Supp Figure 6 [file mmc8.pdf]

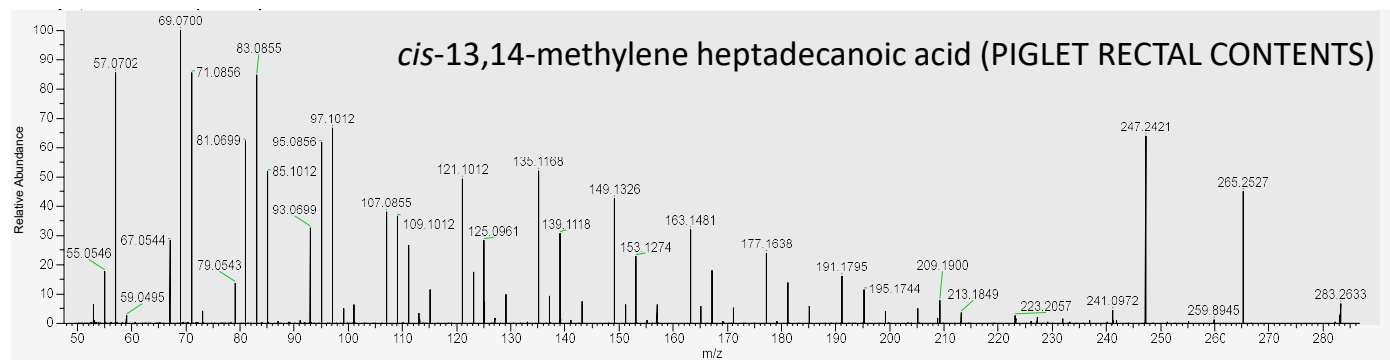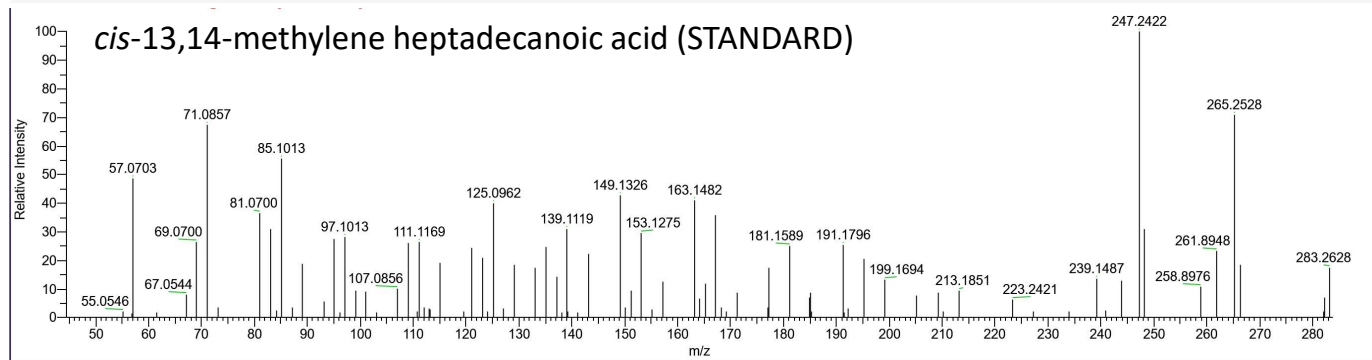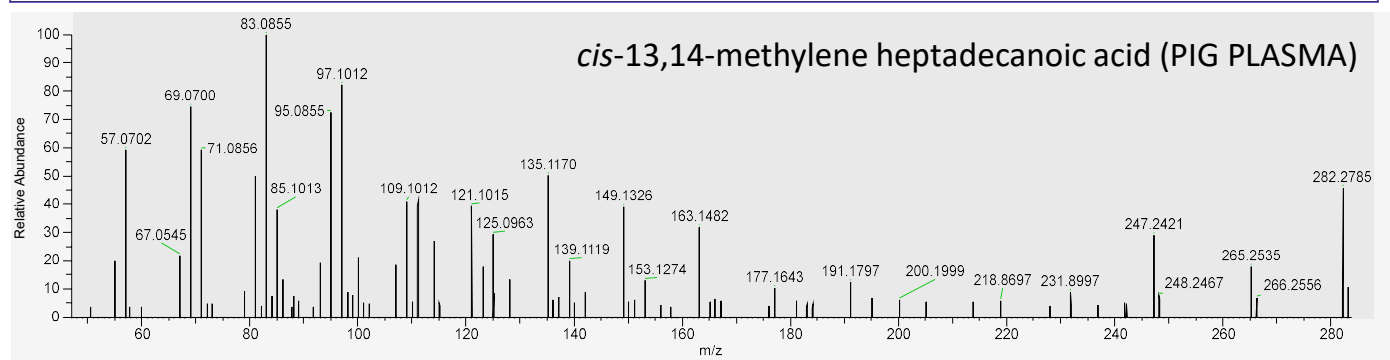

SUPPLEMENTAL FIGURE 7

Supplement: Supp Figure 7 [file mmc9.pdf]
