# Supplementary material for: Novel odd-chain cyclopropane fatty acids: detection in a mammalian lipidome and uptake by hepatosplanchnic tissues
Source: J Lipid Res. 2024 Aug 27;65(10):100632. doi: 10.1016/j.jlr.2024.100632 (PMC11439845; doi:10.1016/j.jlr.2024.100632)
Supplement: Supplemental Materials [file mmc10.pdf]

SUPPLEMENTAL MATERIALS: ***Novel odd-chain cyclopropane fatty acids: detection in a mammalian lipidome and uptake by hepatosplanchnic tissues***
GROWING FEMALE PIG SAMPLING SITE METABOLITE CONCENTRATIONS

sample ID	Pig ID	Port	Site	cis-11,12-Methylene Pentadecanoic Acid Conc. (quantion peak)	cis-13,14-Methylene Heptadecanoic Acid Conc. (quantion peak)	
A1	A	Port1	aorta	4900839	17165667	
A2	A	Port2	portal vein	6636592	19872903	
A3	A	Port3	hepatic vein	3758322	14605116	
A4	A	Port4	femoral vein	5428375	18902426	
A5	A	Port5	renal vein	6791231	25921342	
AG1	AG	Port1	aorta	5011648	18793938	
AG2	AG	Port2	portal vein	6570185	17572351	
AG3	AG	Port3	hepatic vein	4259187	15985010	
AG4	AG	Port4	femoral vein	16520980	36048698	
AG5	AG	Port5	renal vein	14400847	36120853	
AH1	AH	Port1	aorta	3511300	10868606	
AH2	AH	Port2	portal vein	3867375	10386244	
AH3	AH	Port3	hepatic vein			SAMPLE UNAVAILABLE
AH4	AH	Port4	femoral vein	6831919	20353337	
AH5	AH	Port5	renal vein	9837012	22759134	
AI1	AI	Port1	aorta	9524699	23865541	
AI2	AI	Port2	portal vein	7053545	21535150	
AI3	AI	Port3	hepatic vein			SAMPLE UNAVAILABLE
AI4	AI	Port4	femoral vein	9133214	27850432	
AI5	AI	Port5	renal vein	8458715	22410098	
AJ1	AJ	Port1	aorta	3427214	5708704	
AJ2	AJ	Port2	portal vein	3354515	9352449	
AJ3	AJ	Port3	hepatic vein	2644799	5570888	
AJ4	AJ	Port4	femoral vein	3492177	6538121	
AJ5	AJ	Port5	renal vein	4004684	8926111	
AP1	AP	Port1	aorta	2938018	5569412	
AP2	AP	Port2	portal vein	*28618*	*17766*	OUTLIER, not used in flux calculations
AP3	AP	Port3	hepatic vein	1604479	4752836	
AP4	AP	Port4	femoral vein	2810514	6500129	
AP5	AP	Port5	renal vein	1845521	4623806	
AQ1	AQ	Port1	aorta	1964883	6287348	
AQ2	AQ	Port2	portal vein	2227640	4838665	
AQ3	AQ	Port3	hepatic vein	2016120	3999389	
AQ4	AQ	Port4	femoral vein	2409330	4704016	
AQ5	AQ	Port5	renal vein	2264458	5433017	
AR1	AR	Port1	aorta	2467018	6287742	
AR2	AR	Port2	portal vein	2517724	5730294	
AR3	AR	Port3	hepatic vein	2029042	5010940	
AR4	AR	Port4	femoral vein	2704408	6003721	
AR5	AR	Port5	renal vein	3545082	5958212	
AX1	AX	Port1	aorta	7681704	22078426	
AX2	AX	Port2	portal vein	7333928	22847388	
AX3	AX	Port3	hepatic vein	3172798	11928628	
AX4	AX	Port4	femoral vein	5509840	19945760	
AX5	AX	Port5	renal vein	6658235	20679677	
B1	B	Port1	aorta	3758521	14028047	
B2	B	Port2	portal vein	4941218	16683098	
B3	B	Port3	hepatic vein	3421137	11003504	
B4	B	Port4	femoral vein	4639063	12430977	
B5	B	Port5	renal vein	4976589	21440419	
C1	C	Port1	aorta	4124174	18548178	
C2	C	Port2	portal vein	5254781	23639373	
C3	C	Port3	hepatic vein	3013049	14578914	
C4	C	Port4	femoral vein	5939740	23543321	
C5	C	Port5	renal vein	3624240	20703861	
D1	D	Port1	aorta	5123766	18936814	
D2	D	Port2	portal vein	5524789	23671427	
D3	D	Port3	hepatic vein	3259642	15760709	
D4	D	Port4	femoral vein	5326459	21603593	
D5	D	Port5	renal vein	6766850	30692461	
